# ITEXT-BIO: Intelligent Term EXTraction for BIOmedical analysis

**DOI:** 10.1007/s13755-021-00156-6

**Published:** 2021-07-10

**Authors:** Rodrique Kafando, Rémy Decoupes, Sarah Valentin, Lucile Sautot, Maguelonne Teisseire, Mathieu Roche

**Affiliations:** 1 INRAE, Montpellier, France; 2 TETIS, Univ Montpellier, AgroParisTech, CIRAD, CNRS, INRAE, Montpellier, France; 3 CIRAD, F-34398 Montpellier, France; 4 ASTRE, Univ Montpellier, CIRAD, INRAE, Montpellier, France; 5 AgroParisTech, Montpellier, France

**Keywords:** Biomedical terminology, Terminology extraction, Intelligent analysis

## Abstract

Here, we introduce ITEXT-BIO, an intelligent process for biomedical domain terminology extraction from textual documents and subsequent analysis. The proposed methodology consists of two complementary approaches, including free and driven term extraction. The first is based on term extraction with statistical measures, while the second considers morphosyntactic variation rules to extract term variants from the corpus. The combination of two term extraction and analysis strategies is the keystone of ITEXT-BIO. These include combined intra-corpus strategies that enable term extraction and analysis either from a single corpus (intra), or from corpora (inter). We assessed the two approaches, the corpus or corpora to be analysed and the type of statistical measures used. Our experimental findings revealed that the proposed methodology could be used: (1) to efficiently extract representative, discriminant and new terms from a given corpus or corpora, and (2) to provide quantitative and qualitative analyses on these terms regarding the study domain.

## Introduction

The usefulness of terminology extraction from corpora is clearly acknowledged as it has generated a great deal of research and discussion. This well-established process is used in natural language processing and has led to the development of several tailored tools such as TBXTools [31], TermSuite [9], BioTex [22], etc.

Based on [22], our proposal deals with domain-based terminology extraction from heterogeneous corpora, and how to efficiently generate a quantitative and qualitative analysis. To this end, we propose a generic methodology hinged on a combination of extraction and analysis strategies. Term extraction strategies are based on combinations of linguistic, statistical measures, and corpus segmentation approaches, while analysis strategies are based on combinations of extracted terms.

Based on the combined strategies, ITEXT-BIO aims to extract: (1) representative terms, (2) discriminant or relevant terms, and (3) new relevant terms from a corpus or corpora. These strategies are specifically useful for dedicated tasks, such as corpus analysis, specific domain monitoring (e.g. epidemiology) or scientific research monitoring.

This paper is organized as follows. In Section Related work, we briefly present the state-of-the-art related to terminology extraction. Section Dataset description details the dataset dedicated to scientific papers. Sections Methodology and Experiments respectively provide an overview of our proposal and the experiments. In Section Case study: epidemic intelligence, we illustrate the genericity of the proposal by presenting a case study of an implementation of the combined strategies for epidemiological intelligence analysis. We conclude in Section Conclusion by presenting some perspectives for future studies.

## Related work

Domain terminology extraction is a major focus of interest and discussion in natural language processing (NLP) research. It has prompted several proposals of methodologies [20, 32, 34, 36] geared towards effective extraction of terms within a given corpus. Also known as automatic term extraction (ATE), this task is considered in various NLP applications, such as in information retrieval [2, 4, 11, 37], topic modeling [15, 42], domain-based monitoring [1, 19, 27], keyword extraction [7] and summarization [2], ontology acquisition, thesaurus construction, etc.

According to [23], term extraction techniques can be categorized under four approaches: linguistic, statistical, machine learning and hybrid.

Overall, linguistic approaches take morphosyntactic part-of-speach (POS) rules into account to describe terms with common structures [5]. Statistical approaches use statistical measures such as term frequency [35, 43], or term co-occurrence between words and phrases like Chi-square [26]. Machine learning approaches use statistical measures and are mainly jointly focused on term extraction [7, 8, 12], classification [41] and summarization [2]. They combine linguistic and statistic approaches to extract terms from textual data in order to build machine learning models. In [7], the authors highlighted that most of these tasks are tackled with unsupervised learning algorithms. Hybrid approaches include, for instance, C_Value [34], C/NC_Value [13] methods, which combine statistical measures and linguistic based rules to extract multi-word and nested terms. In [6, 30], the authors combine rule-based methods and dictionaries to extract terms from Spanish biomedical texts and specialised Arabic texts respectively.

Studies such as [18, 21] related to these latter approaches have revealed the effectiveness and high performance of hybrid term extraction approaches.

The proposed methodologies apply to several domains. In [22], the authors proposed BioTex, a linguistic and statistical measure-based tool to extract terms related to the biomedical domain. The same approach was used in [1] to detect terms or signals for infectious disease monitoring on the web. In [28], a hybrid methodology was proposed to extract terminology for electronic heath records. This hybrid approach was also adapted by [44] to extract concepts related to Chinese culture.

The overall related studies have focused on techniques and methods for term extraction mainly from corpora. Based on existing methodologies, we oriented our study to develop an efficient approach for term extraction from heterogeneous corpora, along with a set of combined strategies to analyze these terms in the biomedical domain. Our methodology combines and tailors linguistic and statistic criteria associated with structural information in texts in order to highlight relevant terms therein. The presented strategies also aim to overcome the time-consuming issues related to machine learning methods which require manually annotated or partially annotated data.

## Dataset description

Our study focused on the COVID-19 Open Research Dataset^1^ [40] which contains scientific papers on COVID-19 and related historical coronavirus research. Throughout this study, we refer to the dataset as COVID19-MOOD-data.

The COVID19-MOOD-data dataset is divided into two main corpora, respectively named Papers1 and Papers2. Papers1 contains the *commercial use subset (includes PubMed Central content)*, while Papers2 contains the *commercial use subset (includes PubMed Central content), the non-commercial use subset (includes PubMed Central content) and the custom license subset*.

Three data pre-processing operations are performed per corpus (Papers1, Papers2) in order to create three corpora according to the title, abstract and content:

- Title represents the corpus that contains only paper titles;
- Abstract represents the corpus that contains only paper abstracts;
- Content represents the corpus that contains only paper contents.

We named them PapersX-title, PapersX-abstract and PapersX-content, respectively. See Table 1 for further details and Table 2 for the acronym definitions.

**Table 1:** Statistics related to the COVID19-MOOD-data dataset

	$NB_d(C)$	$NB_M (d)$	*std*(*c*)
* Papers1*			
Papers1-title	9315	15	± 8
Papers1-abstract	9315	180	± 94
Papers1-content	9315	4639	± 359
*Papers2*			
Papers2-title	32322	13	± 10
Papers2-abstract	32322	168	± 88
Papers2-content	32322	4913	± 720

**Table 2:** Table legend

Abbreviations	Description
$NB_d(C)$	Number of documents in the corpus
$NB_M(d)$	Average number of words of a document in the corpus
*std*(*c*)	Corpus standard deviation
*NN*	Noun
*NNNN*	Matches singular and plural noun terms
*JJ*	Adjective
*NP*	Proper noun

## Methodology

Here we outline two complementary term extraction and analysis approaches: the free term extraction approach and the driven term extraction approach. The first one is based on a combination of the type of corpus and the statistical measures, while the second is based on a combination of the type of corpus and the morphosyntactic variation rules.

### The free term extraction approach

The free term extraction approach seeks to ensure that users will be able to extract significant terms related to a specific domain from a given corpus. As we mentioned in Section Related work, existing tools have been proposed for term and concept extraction. We opted for the BioTex tool to support the free term extraction mode for several reasons:

- BioTex was initially built for medical domain term extraction.
- BioTex uses hybrid measures (linguistic and several statistical measures) for the term extraction process.
- Most existing tools (e.g. *Maui-indexer*^2^, *Topia Termextract*^3^, *KEA*^4^, etc.) are designed for keyword extraction within single documents, and they only function for English language documents, while BioTex is tailored for terminology extraction and supports sets of documents (corpora) and multi-language use.

Three essential parameters related to the BioTex tool are defined below:

- a corpus: this is the data source from which terms are extracted;
- a statistical measure: as mentioned above, the BioText processing approach is based on linguistic and statistical measures. The linguistic parameter is defined by default, but the user must define the statistical parameter, as several exist, in order to run the term extraction process;
- the number of words to be extracted per concept: so called n-grams, this concerns the length of the extracted terms and ranges from 1 to 4_g for BioTex.

**Fig. 1:**
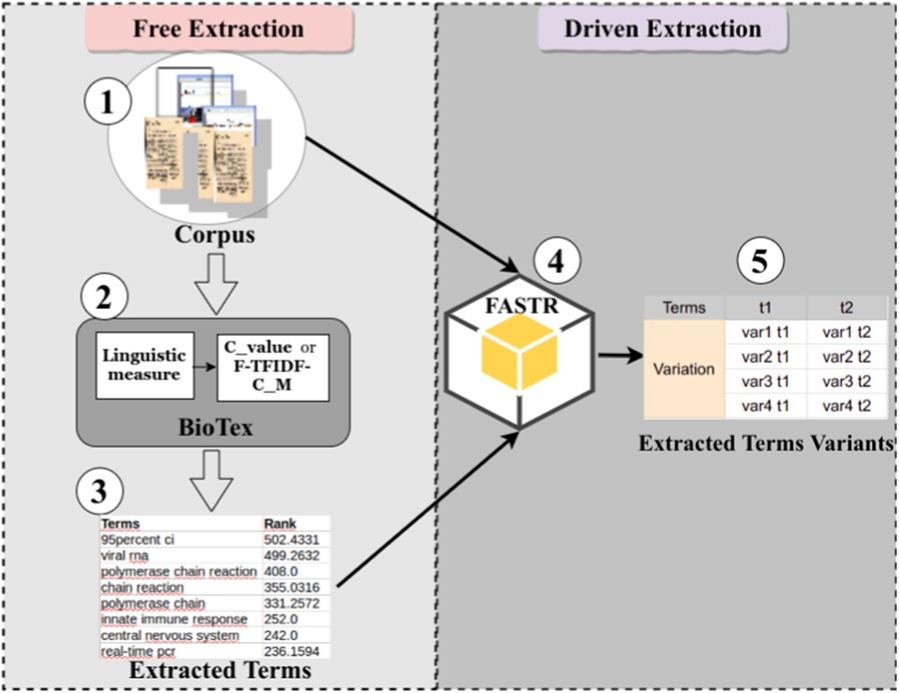
The Free and Driven process for term extraction using BioTex and FASTR

In addition to these parameters, there is the number of linguistic patterns (like NN NN, JJ NP NP, NN NP NP, etc.) that can be associated, but this is preset at 20 by default in BioTex. BioTex also includes patterns for verb terms, such as: NN VBD NN NN, NP NN VBD NN NP, etc. Figure 1 outlines the overall three-step process for free term extraction.

At the end of the BioTex process, extracted terms are classified in two sets: TermSet, which only contains single word terms (SWT), and MultiTermSet, which contains multi-word terms (MWT). By using the Driven Extraction process (with FASTR), we can capture the entire term for a given incomplete one obtained during the first step (Free Extraction). The Driven Extraction process step uses incomplete terms to capture the entire terms in the document. For example, if “higher risk acute” or “higher risk area” terms are extracted in the Free Extraction process step, an entire term which could be“higher risk acute care area” will be obtained during the Driven Extraction process.

### The driven term extraction approach

This extraction approach seeks to ensure that the terms extracted using BioTex could be used to improve the domain terminology. From a given term, the process aims to extract some variations of this term that exist in the corpus.

The overall processing under this approach is handled with FASTR [17]. FASTR is a rule-based linguistic tool that generates morphosyntactic variants of terms. We respectively note *NN, NNS, NNP, NNPS* for noun paterns, *VB, VBD, VBG, VBN, VBP, VBZ* for verbs, *RB, RBR, RBS* for adverbs and finally *JJ, JJR, JJS* for adjectives. It enables extraction of variants of a given term in full-text documents. For a given term, FASTR helps extract nearby or long terms that contain the initial term. Figure 1 illustrates the two steps (4 and 5) of the driven term extraction approach. For a given term, FASTR helps extract nearby or long terms that contain the initial one.

The driven process has the advantage of extracting relevant new terms that BioTex cannot extract from the corpus.

### Proposed combination for term extraction

**Fig. 2:**
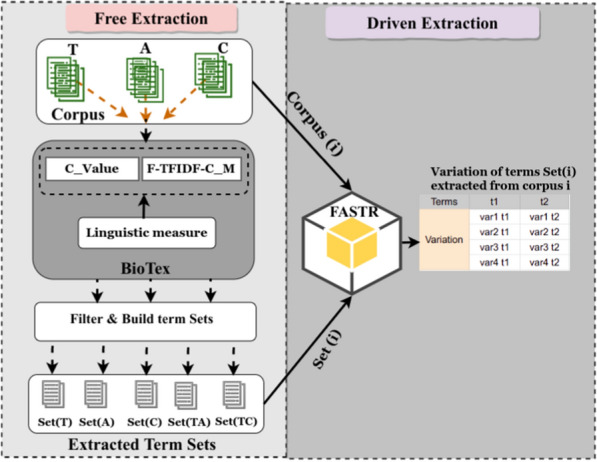
Proposed combination for term extraction

Based on the elements given in Sects. The free term extraction approach and The driven term extraction approach, we propose a workflow in Fig. 2 for term extraction and analysis dedicated to scientific papers. We outline this workflow according to the type of corpus, measure, and approach:

- *The type of corpus*: as described in the data section, for a given paper, we considered three parts to build the corresponding corpora, i.e. the Title (T), Abstract (A) and Content (C);
- *The measures*: BioTex integrates several statistical measures, each of which uses a specific strategy to compute the term score. In this case, we selected the two measures C_Value and F-TFIDF-C_M. C_Value indicates the importance of terms that appear most frequently in a document, based on the idea that the frequency of appearance of a term in the document reflects its importance in the document. Moreover, based on frequency criteria, C_Value favors multi-word term extraction by taking into account nested terms (e.g. virus) in multi-word terms (e.g. influenza virus) [13]. F-TFIDF-C_M represents the harmonic mean of the two C_Value and TF-IDF values, which ranks terms by weight according to their relevance in the document while taking the whole corpus into account [24]. C_Value and F-TFIDF-C_M are complementary, as the first favors relevant MWT extraction while the second gives weight to discriminant terms. For each measure, the aim is to organise the extracted terms in to five sets. (1) Terms corresponding to the Title corpus Set(T), (2) terms corresponding to the Abstract corpus Set(A), (3) terms corresponding to the Content corpus Set(C), (4) terms that intersect within the Title and the Abstract corpus Set(TA), and (5) terms that intersect within the Title and the Content corpus Set(TC).
- *The approach*: terms could be extracted using both a given corpus and a specific statistical measure in a free extraction approach. Moreover, for the driven process, term variations are extracted by using both a given corpus and specific set of terms. The set of terms could be defined from the output of the previous approach.

## Experiments

To set the parameters, throughout our study we used C_Value and F-TFIDF-C_M as statistical measures, 50 different patterns or term extraction rules, and a number of words ranging from 1 to 4-g ($n = 1,2,3,4$). These parameters are applied for corpora described in section 3. The choice of C_Value and F-TFIDF-C_M is based on the findings of previous studies [13, 24] which showed that both allow efficient SWT and MWT extraction.

Before applying BioTex, some specific pre-processes were applied for the Papers1-content and Papers2-content corpora due to their size. Papers1-content was divided into 09 sub-corpora (8 corpora of 1000 documents each and 1 corpus of 1315 documents) and Papers2-content into 32 sub-corpora (31 corpora of 1000 documents each, and 1 corpus of 1332 documents). Each corpus was partitioned into smaller units to enhance scalability. The results obtained from the smaller units were then composed by computing the average ranked values. The final rank for a given term was thus equal to the average of its ranked values in all sub-corpora in which it was present. The final result gave a set of terms, listed in ascending order according to the ranking values.

Table 3 shows an example of the MWT set obtained using BioTex. The *Terms* column contains the extracted terms, the *in_umls* column indicates if the corresponding term is available in the Unified Medical Language System (UMLS) Metathesaurus [3] or not, and *rank* shows the significance of the term based on statistical measures in the whole list of terms for a given corpus. In our study, we used the UMLS Metathesaurus as reference for the extracted terms as our study is linked to a biomedical terminology analysis. This comparison aimed to separate new terminologies or terminologies that were not yet listed in the Metathesaurus.

**Table 3:** Example of BioTex ouput

Terms	in_umls	Rank
Public health	1	1602.3971
Respiratory syndrome	0	1481.9399
Infectious disease	1	1198.2317
Virus infection	1	1126.9083
Influenza virus	1	1023.8858
Immune response	1	1008.0362

### The free term extraction approach

We used BioTex, as outlined in Section The free term extraction approach, to extract terms from corpora in free mode. Several analyses are performed below on the obtained results. To this end, we conducted the experiments to address three main questions: (1) for each corpus, what are the most representative terms or domain concepts (terms that summarize the main content of the corpus) per statistical measure? (2) for each corpus, what are the most representative concepts for both measures? and (3) what are the discriminant and common concepts of the overall corpus?

For each case, we determined if the extracted terms exist or not in the UMLS Metathesaurus.

#### Corpus representative terms

In this section, we illustrate how representative terms can be extracted from different datasets. Based on the BioTex ranking measures, a term is more important than another one in a given corpus if it has a higher ranking than the other term.

**Fig. 3:**
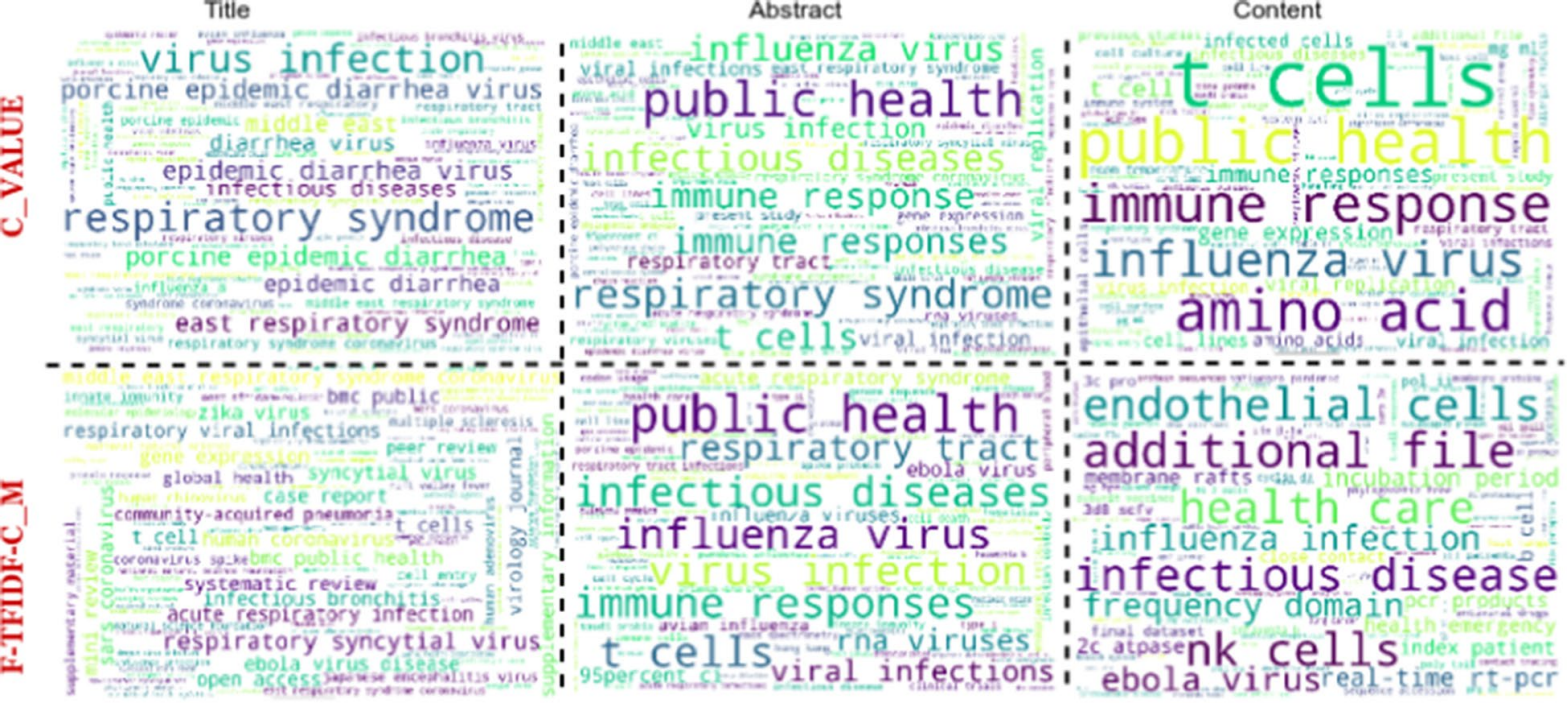
Representative terms from Papers1

Figure 3 shows representative terms for the Title, Abstract and Content corpora with the corresponding statistical measures (see Tables 7 and 8 for more details).

This figure highlights which terms are important in each part of the Papers. Note that the extracted terms are different for each measure and sub-corpus, but some of them are similar for both. For example, terms like *public health, immune responses* are extracted using both measures from the Abstract corpus.

**Fig. 4:**
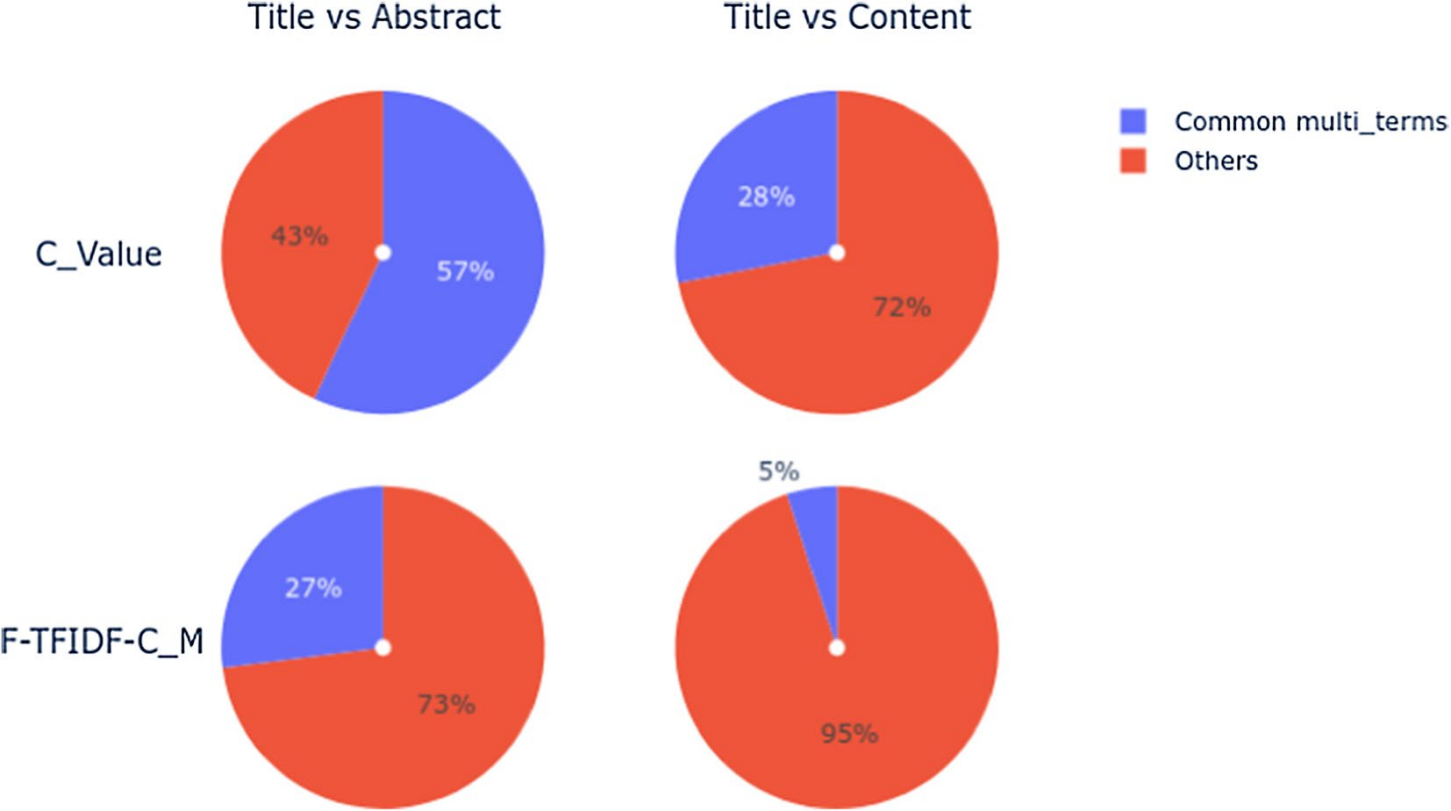
Common terms in Papers2

In order to quantitatively display the number of representative intersecting terms from different corpora, we show common terms between Title vs Abstract, and Title vs Content corpora for the Papers2 corpus in Fig. 4. For both measures, Title terms are more representative in the Abstract than in the Content of Papers, i.e. 57% and 27% compared to 28% and 5%, respectively, for Title vs Abstract and Title vs Content. However, we noted that terms extracted with C_Value generated more common terms than those extracted with F-TFIDF-C_M. The common terms represent terms extracted at once in the Title, Abstract and Content corpus for each measure.

As indicated, extracted terms were compared with the UMLS Metathesaurus. Table 4 shows the TOP@20 terms extracted for the Papers1-content corpus using C_Value and F-TFIDF-C_M measures. Bold terms are not in the UMLS Metathesaurus.

**Table 4:** TOP@20 terms extracted from Paper1-content using C_Value and F-TFIDF-C_M - SWTs vs MWTs

C_Value Measure					
	SWTs				
TOP 20	Cells	Virus	Infection	Protein	Study
	Data	**figure**	al	Patients	Expression
	rna	Analysis	Result	Disease	p
	Mice	c	**samples**	Influenza	Number
	**MWTs**				
TOP 20	t cells	Public health	Amino acid	Immune response	Gene expression
	Viral replication	**infected cells**	Cell lines	Viral infection	Virus infection
	mg ml	Infectious diseases	** Present study**	Respiratory tract	Epithelial cells
	**Previous studies**	Room temperature	Cell culture	**Additional file**	Viral infection
**F-TFIDF-C_M Measure**					
	**SWTs**				
TOP 20	Mice	Patients	Influenza	Proteins	Health
	dna	Vaccine	Transmission	Research	Model
	Children	Outbreak	Vaccination	e	China
	Peptide	Fusion	Network	Percent	mers-cov
	**MWTs**				
TOP 20	**Additional file**	Infectious disease	nk cells	Health care	Endothelial cells
	**Frequency domain**	Ebola virus	**Influenza infection**	**Real-time rt-pcr**	Incubation period
	**Health emergency**	**Index patient**	**Membrane rafts**	**pcr products**	**2c atpase**
	b cell	**Close contact**	**Final dataset**	**3d8 scfv**	pol ii

In bold terms not in the UMLS thesaurus

According to these TOP@20 terms, we can see that:

- the majority of the SWTs are in the UMLS Metathesaurus for both statistical measures (C_Value or F-TFIDF-C_M);
- for MWTs, several terms are not in the UMLS Metathesaurus. These terms can be categorized as:
- *UMLS sub-terms* these are terms that do not exactly match to those present in the UMLS Metathesaurus but could be part of them. For example, *health emergency* is part of terms like *Emergency Health Services* in the UMLS Metathesaurus;
- *New terms* these terms are not in the UMLS Metathesaurus, but are meaningful (or not) in the COVID-19 context. For example, terms like *close contact* relate to the COVID-19 contagion mode.

**Fig. 5:**
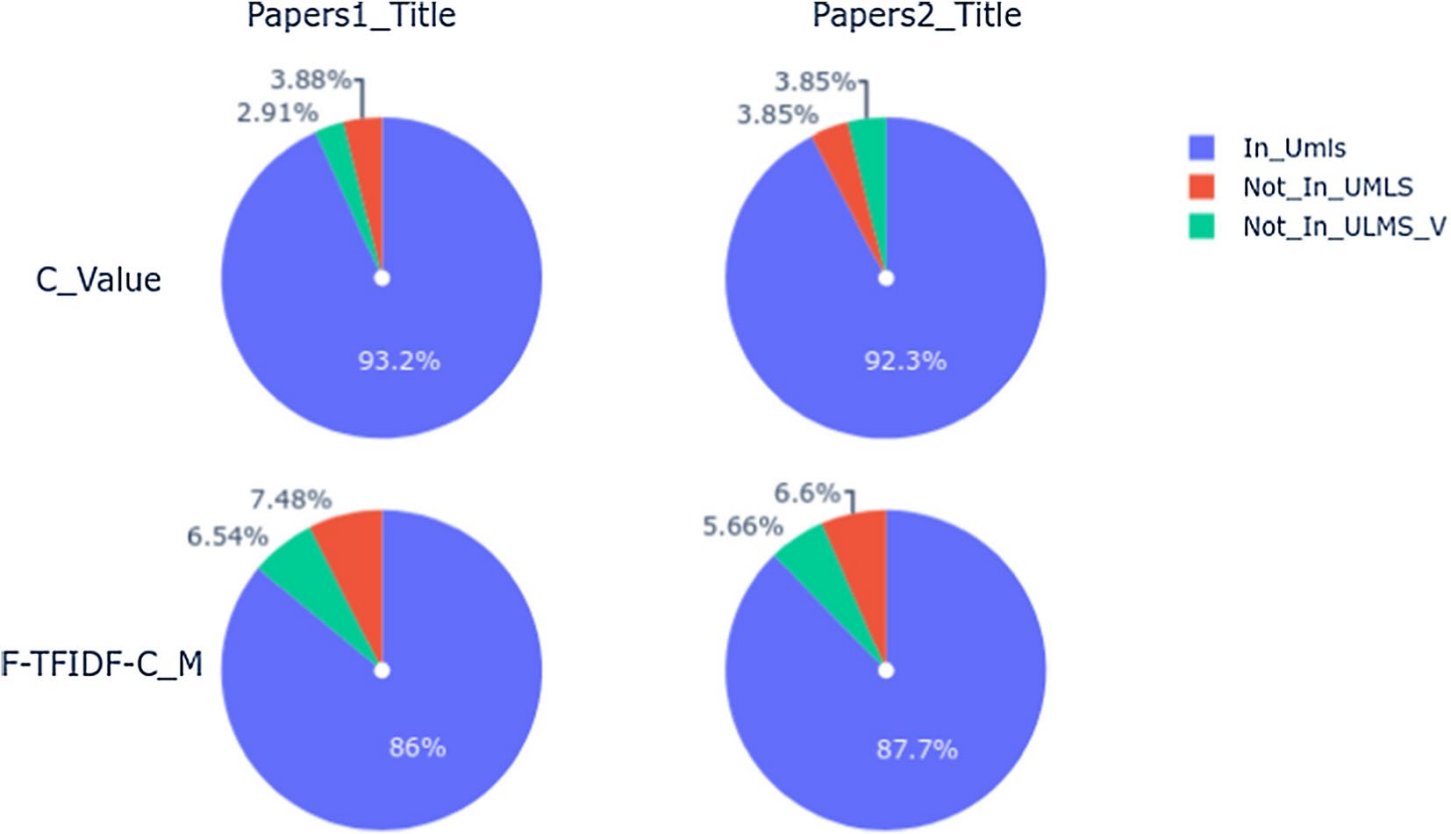
C_Value vs F-TFIDF-C_M SWTs

**Fig. 6:**
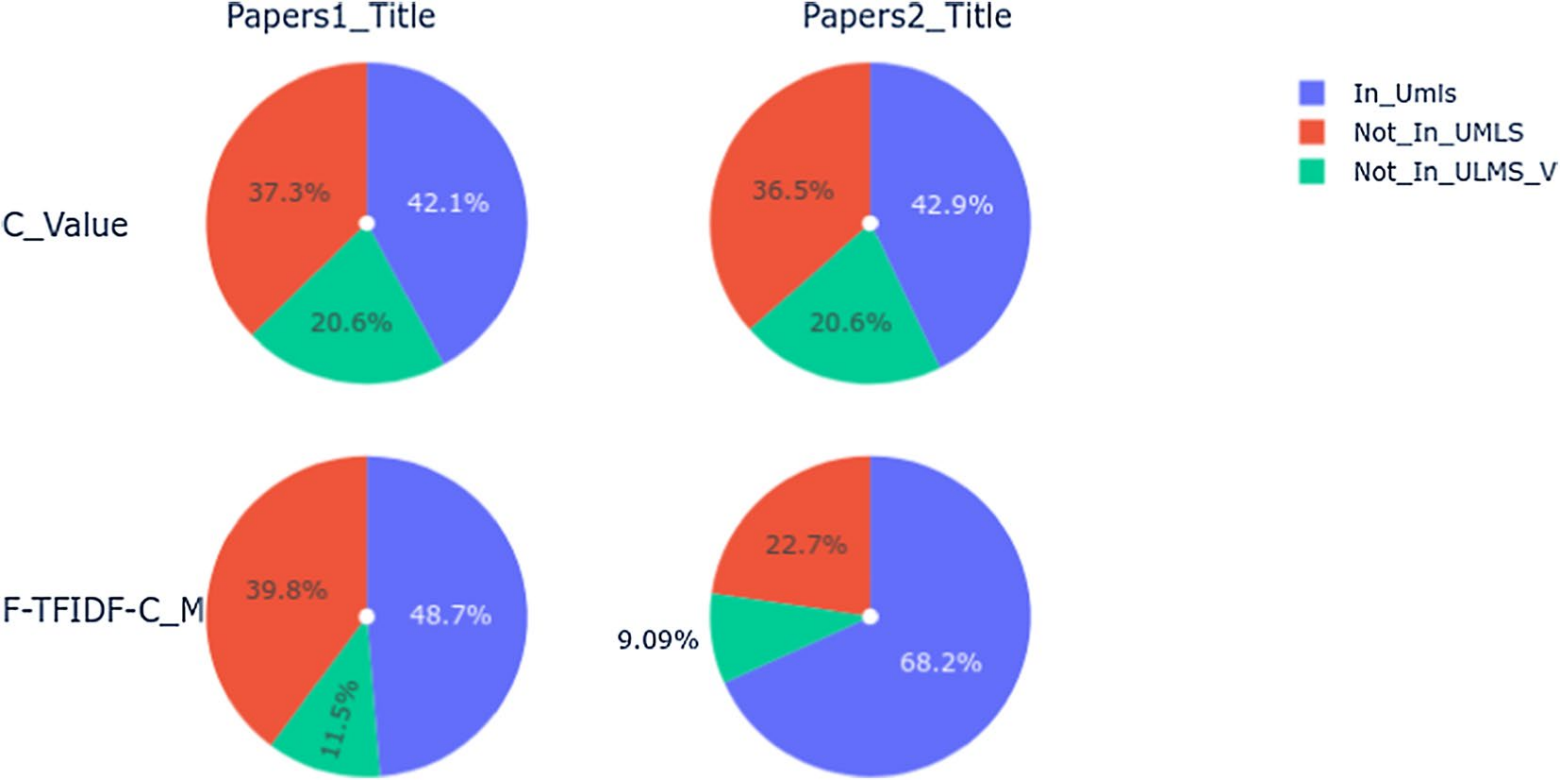
C_Value vs F-TFIDF-C_M MWTs

Figures 5 and 6 illustrate the number of terms out of the TOP@100 terms (in percentage) for each measure (C_Value, F-TFIDF-C_M) and dataset (Papers1-title, Papers2-title):

- In_UMLS: the number of terms in the UMLS Metathesaurus;
- Not_In_UMLS_V: the number of terms that do not exactly match the UMLS terms, but have some variants or are part of the UMLS terms;
- Not_In_UMLS: the number of terms that do not match the UMLS terms at all. We indicate these as new terms. Terms which are not in the UMLS Metathesaurus but which could have greater meaning in the study context or which could be added to the UMLS Metathesaurus.

According to these statistics, we first note that the C_Value and F-TFIDF-C_M measures enable extraction of more conventional terms or terms in the UMLS Metathesaurus regardless of the corpus. Secondly, we note that F-TFIDF-C_M generates more new terms (Not In UMLS) than C_Value regardless of the corpus. Finally, the number of new terms is more substantial with MWTs (Fig. 6) than SWTs (Fig. 5) regardless of the measure.

#### Relevant term extraction from corpora for both measures

This involves quantitative and qualitative analysis of the terms extracted within each corpus, while taking both measures (C_Value and F-TFIDF-C_M) into account. In other words, it consists of analysing terms obtained for both measures, i.e. terms detected at the same time, and also terms specific to each of them.

The quantitative analysis aims to highlight, for each dataset, the number of terms obtained by each measure, the number of terms obtained for both measures, and which are available or not in the UMLS Metathesaurus. While the qualitative measure aims to highlight, in each case, how the terms obtained are important or not regarding the study domain.

For the data representation, we take advantages of Venn Diagram [16], see in Appendix Fig. 10 the distribution of the Papers2-title corpus terms. Terms are organised in different sections. For example, *gene expression, human coronavirus, case report, public health, respiratory syncytial virus, etc.* are available in UMLS Metathesaurus and are recognized by both measures (C_Value and F-TFIDF-C_M). According to the study domain, these terms will tend to be more representative and important in the whole corpus. Moreover, for each measure there are new terms which are not in the UMLS Metathesaurus.

#### Discriminant and common term extraction from corpora

In this case, term analysis is performed per dataset or by jointly considering multiple corpora, i.e. between Title, Abstract and Content corpora. Appendix Fig. 11 corresponds to discriminant and common term extraction from Papers1-title, Papers1-abstract and Papers1-content.

There are common terms in the overall corpus such as *gene expression, virus replication, influenza virus, etc.*. These terms tend to be relevant in the Title, Content and Abstract corpora.

Moreover, *[respiratory infection, acute respiratory infection, etc.]*, *[innate immune response, endoplasmic reticulum, etc.]*, and *[nucleotide sequences, room temperature, etc.]* are discriminant terms in the Title, Abstract and Content corpora.

### The driven term extraction process

We performed a driven term extraction strategy using FASTR. Our proposal addresses two main questions: (1) For a given set of terms, how can new and relevant terms variants be extracted from a corpus based on the terms? (2) Do some of the new terms exist in the UMLS Metathesaurus? In our experiment, we used the common terms extracted in section 5.1.3 based on the fact that they were more representative and relevant throughout the corpora.

**Fig. 7:**
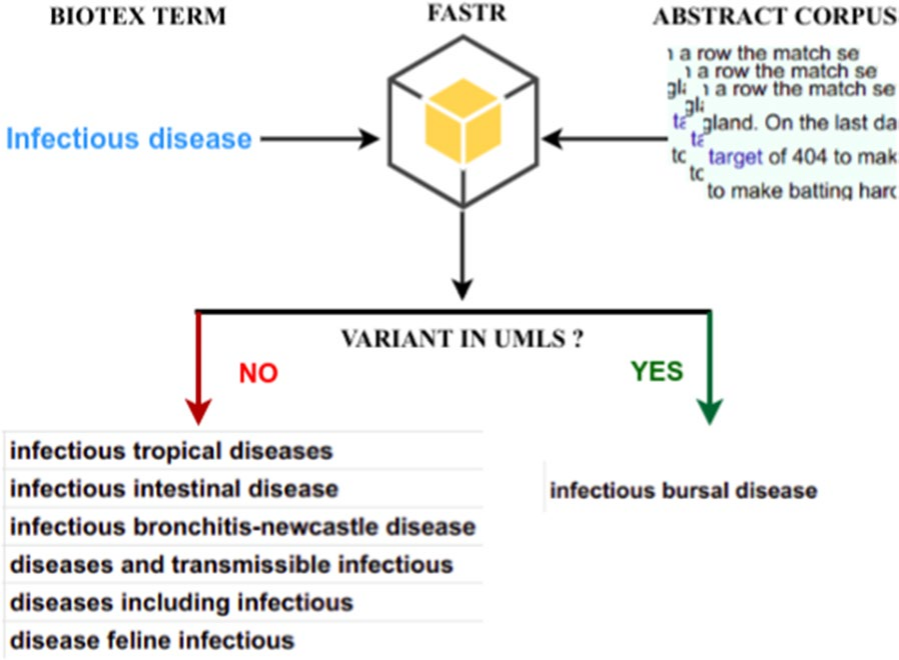
Example of term variants

Figure 7 shows an example of variant terms extracted with the term *infectious disease*. Among these variants, we only show those which are not in the UMLS Metathesaurus since they are new and might be more informative.

Table 5 contains a list of TOP@10 variants extracted with six initial terms. Among them, we highlighted (in bold) terms matching terms in the UMLS Metathesaurus. Like free mode extraction, term variant mode may be used to extract useful terms.

**Table 5:** Term extraction variations using FASTR

**Terms**	Infectious disease	Virus replication	Laboratory tests	Respiratory syndrome	Preventive measure	Syndrome coronavirus
**Variations**	Diseases including infectious	**Replication competent viruses**	Laboratory confirmation tests	**Respiratory distress syndrome**	**Preventive measures**	Syndrome coronavirus-related coronavirus
	Infectious pulmonary diseases	replication of N1347A virus	**Laboratory testing**	Respiratory acute syndrome	Preventive hygienic measures	Syndrome human coronavirus
	**Infectious bursal disease**	Virus optimal replication	Testing presents isolation laboratories	Syndrome coronavirus and respiratory	Prevention community-engaged measures	Syndromic Surveillance Coronavirus
	**Infectious lung diseases**	Replicating influenza viruses	Laboratory diagnostic testing	Respiratory tract syndromic	Preventive health measures	Syndrome virus coronavirus
	Infectious acute disease	Replication of human viruses	Laboratory genomic testing	Respiratory insufficiency syndrome	Preventive behavioral measures	Coronavirus Associated Syndromes

Terms in the UMLS Metathesaurus in bold

### Combined strategies for term analysis

Combined strategies for term analysis concern two levels: (1) Intra-corpus term extraction, and (2) Inter-corpus term extraction.

**Fig. 8:**
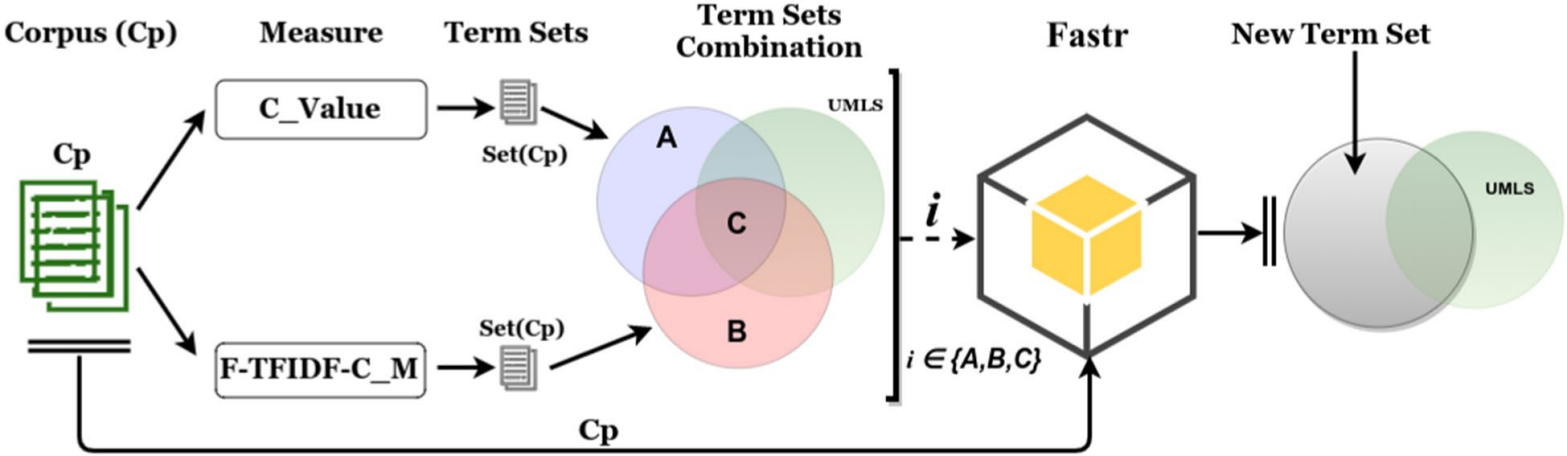
Combined intra-corpus term extraction strategies

**Fig. 9:**
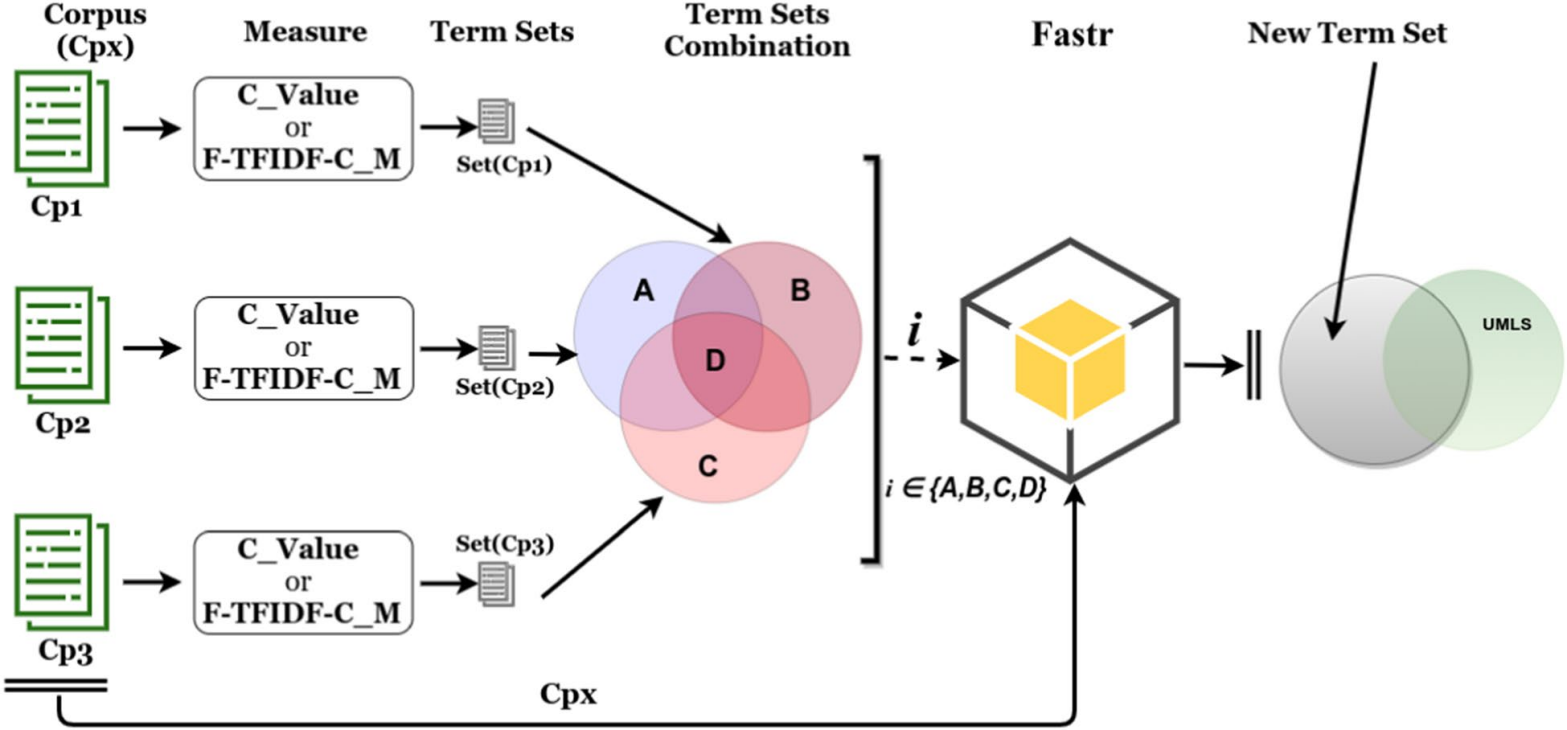
Combined inter-corpus term extraction strategies

- Combined intra-corpus term extraction strategies: these are geared towards extracting common or discriminant terms from a given corpus. To this end, extracted terms from both measures are compared. We show the process in Fig. 8, where the set of terms Set(Cp) extracted from the corpus Cp (Title, Abstract or Content) using each measure (C_Value, F-TFIDF-C_M) are jointly compared with the UMLS Metathesaurus terms. Set A represents corpus terms specifically extracted with C_Value, set B represents terms that are specific to F-TFIDF-C_M, while set C represents common terms from both measures and UMLS Metathesaurus elements. We consider that sets A and B are discriminant terms of the corpus according to the measures, and otherwise set C is considered as containing common terms or the most representative terms of the corpus. The new term extraction process with FASTR is run with one of the combined sets (discriminant or common) and the corpus.
- Combined inter-corpus term extraction strategies: these are geared towards extracting common and discriminant terms, while taking several corpora into account for a given measure. As illustrated in Fig. 9, for each measure (C_Value or F-TFIDF-C_M), the sets of terms Set(Cp1), Set(Cp2), Set(Cp3) are extracted respectively from corpus Cp1 (Title), Cp2 (Abstract), and Cp3 (Content). These sets are compared in order to compute the common term set D for both corpora, and discriminant term sets A, B, C, respectively, for corpora Cp2, Cp1 and Cp3. In this context, new terms are extracted using one of the combined sets with one corpus (Cpx).

## Case study: epidemic intelligence

Epidemic intelligence (EI) aims to detect, investigate and monitor potential health threats in a timely manner [33]. In addition to conventional surveillance system monitoring, such as outbreak notifications from the World Organisation for Animal Health (OIE), the EI process increasingly mainstreams unstructured data from informal sources such as online news. Several web-based surveillance systems have been developed and used to support public health and animal health surveillance (ProMED [25], HealthMap [14], GPHIN [29], PADI-web [38], etc.). In this case study, we focused on the choice keywords with the PADI-Web system for COVID-19 surveillance (i.e. driven surveillance) and for monitoring unknown diseases (i.e. syndromic surveillance).

The Platform for Automated extraction of Disease Information from the web (PADI-web^5^) is an automated surveillance system for monitoring the emergence of animal infectious diseases, including zoonoses [1, 38]. PADI-web monitors Google News through specific really simple syndication (RSS) feeds, targeting diseases of interest (e.g. African swine fever, avian influenza, etc.). PADI-web also uses unspecific RSS feeds, consisting of combinations of symptoms and hosts (i.e. species), thus allowing syndromic surveillance and detection of unusual disease events. RSS feeds consists of combinations of different categories of terms (i.e. keywords) including symptoms, disease names and species.

PADI-Web has been used for monitoring COVID-19 disease [39]. In this context, the choice of COVID-19 surveillance terms is crucial.

In the following subsections, we discuss the choice of terms given by ITEXT-BIO to use in the PADI-Web system [38] and other web-based surveillance systems [14, 25, 29] for COVID-19 and syndromic surveillance. This enables evaluation of the relevance of terms generated by our approach for a dedicated task, i.e. web-based health surveillance.

### Relevant term extraction

We compared the relevance of the top 10 terms extracted from Papers2 corpora with either C_Value or F-TFIDF-C (Table 6). Table 9 gives more details on these terms. The relevance was assessed by classifying the terms in one or more of the following categories:

- COVID-19 surveillance: epidemiological terms specific to COVID-19 (e.g. *coronavirus spike*).
- Syndromic surveillance: epidemiological terms not specific to a particular disease (e.g. *infectious bronchitis*).
- Domain relevant: terms related to health, i.e. either to specific diseases (e.g. *porcine epidemic diarrhoea*) or unspecific (e.g. *virus infections*). The Domain relevant category thus includes the two previous categories, plus diseases other than COVID-19.
- Part of disease multiword expression (MWE): part of a multiword expression corresponding to a disease name (e.g. *East respiratory syndrome* for *Middle East syndrome coronavirus*).

Among the terms extracted with C_Value from Titles, Abstracts or Titles and Abstracts, six to seven were parts of disease MWE. Only one term extracted with F-TFIDF-C_M was a part of disease MWE. C_Value could thus be of particular interest for extracting disease name variants, even if they are incomplete. For domain relevant COVID-19 surveillance and syndromic surveillance terms, F-TFIDF-C_M obtained better results than C_Value, even when the frequency of relevant terms was low (from one to five out of ten terms). No common terms were extracted from (Title + Abstract) or from (Title + Content) using F-TFIDF-C_M. Using C_Value, only three common terms were extracted from Title + Content. Among the top 10 terms extracted from Title + Abstract with these metrics, seven were parts of disease MWE. Regardless of the term category, we extracted more relevant terms from Titles and Abstracts than from Contents. This is in line with the fact that Title and Abstracts are more rich in key information and relevant terms due to their length limitation.

**Table 6:** Relevance of terms extracted from Papers2 depending on the metrics (C_Value or F-TFIDF-C_M)

Corpus (Papers2)	Measure	**n**	Domain relevant	COVID-19 surveillance	Syndromic surveillance	Part of disease MWE
Title	C_Value	10	3	0	2	6
Title	F-TFIDF-C_M	9	4	1	1	1
Abstract	C_Value	10	1	0	0	6
Abstract	F-TFIDF-C_M	10	5	1	2	1
Content	C_Value	10	0	0	0	1
Content	F-TFIDF-C_M	10	2	0	2	0
Title + abstract	C_Value	10	3	0	0	7
Title + abstract	F-TFIDF-C_M	0	-	-	-	-
Title + content	C_Value	3	1	0	0	2
Title + content	F-TFIDF-C_M	0	-	-	-	-

### Driven term extraction

We selected terms extracted in Section 6.1: *respiratory tract, viral infections, SARS coronavirus, incubation period, influenza virus, respiratory infections and infectious diseases*. We randomly extracted the variants with FASTR (Section The driven term extraction approach). An epidemiologist manually evaluated the relevance of 10 randomly selected variants per term. Among the 60 evaluated terms (see Table 10), 72% (43/60) were relevant and 7% (4/60) were irrelevant. For 13 variants (22%), the relevance could not be assessed because the expression was truncated and ambiguous, such as “disease has an infectious” for the term “infectious diseases”. FASTR thus seems to be an effective tool for generating term variants efficiently. However, we noted that FASTR generated up to 774 variants for a single term. Thus, to avoid random selection of terms, it would be interesting to compute a relevance index that could be used to rank the proposed variants. Besides, several extracted variants were fragments of expressions that could not be evaluated. This issue could be overcome by displaying the variant context (i.e. the sentence in which the variants appeared).

## Conclusion

In this paper we describe ITEXT-BIO, a generic methodology for biomedical term extraction. We show how it allows users to extract terms (or concepts) from different types of textual data using several combined strategies. The free term extraction approach extracts terms from corpora, while the driven term extraction approach extracts, from a corpus and a set of terms, a set of variations of these terms.

We illustrate that the proposed combined strategies based on statistical measures and textual segments help efficiently extract and categorize terms (representative, discriminant and new terms) from a corpus or corpora. We also quantitatively and qualitatively analysed the extracted terms to determine those related to the study domain and those that could be considered as emerging terminology for disease monitoring.

Our future studies will focus on term extraction and analysis by: (i) taking different sections of papers into account and applying the methodology to different types of corpora derived from newspapers or social media such as Twitter, (ii) considering combinations of tools other than BioTex, and (iii) introducing word embedding strategies like BERT [10] to capture semantic aspects of the extracted terms in order to reduce context ambiguity.

## Figures and Tables

**Fig. 10:**
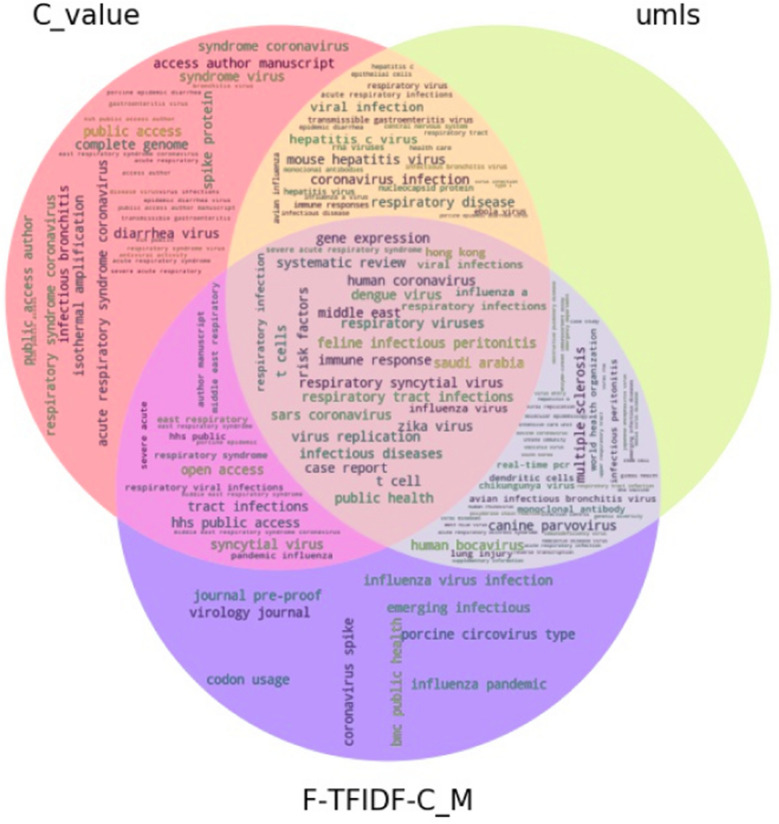
Distribution of concepts according to the measures and their presence in the UMLS Metathesaurus: from Papers2-title corpus

**Fig. 11:**
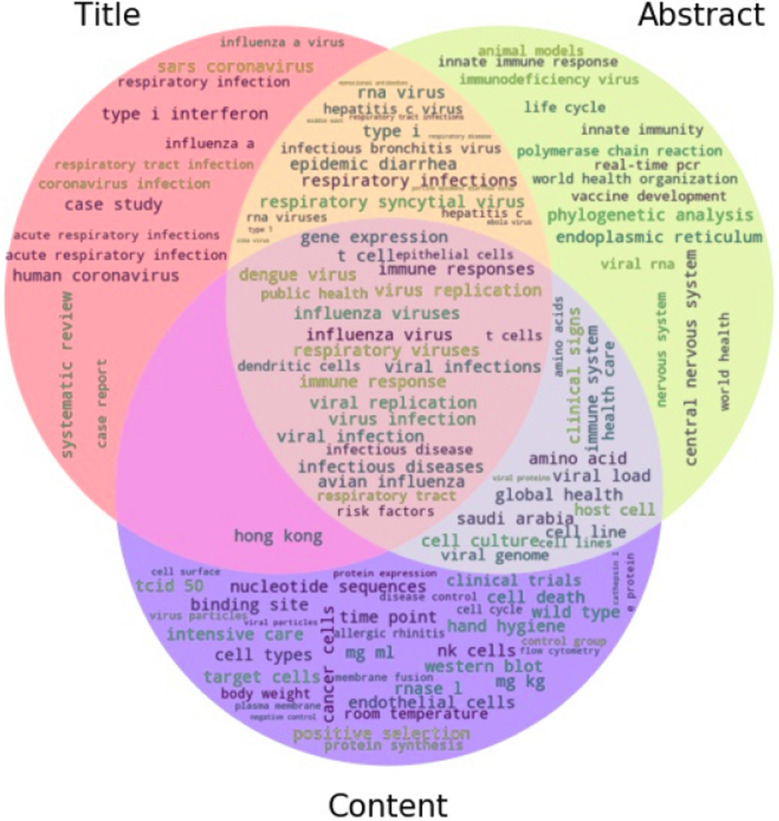
Distribution of representative concepts when taking multiple corpora into account using C_Value: Papers1 corpora

## Appendix A

See Tables 7, 8, 9, and 10.

**Table 7:** Best ranked terms extracted from Paper1 using F-TFIDF-C_M

F-TFIDF-C_M					
Title Corpus		Abstract Corpus		Content Corpus	
terms	rank	terms	rank	terms	rank
Respiratory syncytial virus	1.9880	Public health	1.9986	Additional file	1.9976
Middle east respiratory syndrome coronavirus	1.9846	Infectious diseases	1.9979	Infectious disease	1.997
Systematic review	1.9842	Immune responses	1.9976	nk cells	1.997
Open access	1.9819	Influenza virus	1.9976	Health care	1.996
Zika virus	1.9819	t cells	1.9975	Endothelial cells	1.9957
Gene expression	1.9795	Virus infection	1.9974	Frequency domain	1.9957
Virology journal	1.9788	Respiratory tract	1.9973	Ebola virus	1.9948
Human coronavirus	1.976	Viral infections	1.9969	Influenza infection	1.9943
Case report	1.9756	RNA viruses	1.9967	Real-time rt-pcr	1.9933
Syncytial virus	1.9752	Acute respiratory syndrome	1.9961	Incubation period	1.99325
t cell	1.9746	95percent ci	1.996	Health emergency	1.9932
Infectious bronchitis	1.9726	Ebola virus	1.9945	Index patient	1.9932
Sars coronavirus	1.9723	Influenza viruses	1.9943	Membrane rafts	1.9931
BMC public health	1.9701	Avian influenza	1.9939	pcr products	1.9929
t cells	1.9689	Respiratory tract infections	1.9938	2c atpase	1.9926
Acute respiratory infection	1.9672	Health care	1.9925	b cell	1.9924
Mini review	1.9636	Hepatitis c	1.9922	Close contact	1.9924
Respiratory viral infections	1.9636	Type I	1.9918	Final dataset	1.9922
BMC public	1.9625	Cell line	1.9914	3d8 scfv	1.9921
Ebola virus disease	1.9592	Spike protein	1.9909	Pol ii	1.992
Supplementary information	1.9574	Codon usage	1.9908	3c pro	1.992
Community-acquired pneumonia	1.9543	Pandemic influenza	1.9907	Influenza pandemic	1.9919
Global health	1.9543	Endoplasmic reticulum	1.9904	Phylogenetic tree	1.9918
Peer review	1.9543	Saudi Arabia	1.9904	Protein vi	1.9917
Japanese encephalitis virus	1.9512	Innate immunity	1.9903	ag nps	1.9916
Innate immunity	1.9488	Porcine epidemic	1.9903	Influenza b	1.99125
Multiple sclerosis	1.9488	Global health	1.9902	ifn $\beta - 1\alpha$	1.991
Human rhinovirus	1.9466	Vaccine development	1.9901	ill patients	1.9908
Supplementary material	1.9442	Cell death	1.9898	Poly tail	1.9908
Cell entry	1.9417	Infectious disease	1.9896	Host range	1.9906
Coronavirus spike	1.9417	Peripheral blood	1.9895	Cyclin d3	1.9903
Human adenovirus	1.9417	Hong Kong	1.9894	Sequence accession	1.9903
East respiratory syndrome coronavirus	1.9414	Immune cells	1.9888	Antiviral drugs	1.9897
Mers coronavirus	1.9388	Cell cycle	1.9886	Subunit vaccines	1.9897
West Africa	1.9388	Clinical trials	1.9885	Protein sequences	1.9895
Molecular epidemiology	1.9323	Infection control	1.9884	Oil spill	1.9895
National natural science	1.931	Mass spectrometry	1.9883	Swine flu	1.9894
Natural science foundation	1.931	Genome sequence	1.9881	Membrane proteins	1.9893
Rift valley fever	1.931	Clinical samples	1.9877	Contact tracing	1.9891
National natural science foundation	1.9307	Acute respiratory infections	1.9874	sars 3a	1.9889
Influenza infection	1.9284	Severe disease	1.9868	Critical care	1.9888
Protein response	1.9284	Hepatitis b	1.9864	hk-2 cells	1.9888
Science foundation	1.9284	Host response	1.9864	ap2 group	1.9887
Supplementary materials	1.9284	Type II	1.9864	prp sc	1.9887
Natural science	1.9241	Nucleic acids	1.9862	t-cell responses	1.9887
Respiratory syndrome coronavirus infection	1.9241	Surveillance systems	1.9859	DNA vaccines	1.9886
Influenza virus	1.9212	Influenza virus infection	1.9852	Reverse genetics	1.9886
Obstructive pulmonary disease	1.92	Antiviral drugs	1.9851	Health system	1.9884
Emerging microbes	1.9193	DNA vaccine	1.9847	$b7-h1$	1.9884
Original research	1.9193	Influenza infection	1.9845	hcv infection	1.9883
Retrospective study	1.9193	Reference genes	1.9842	Lung cancer	1.9879
Phylogenetic analysis	1.9153	Cell types	1.984	Nucleocapsid protein	1.9879
Respiratory syndrome coronavirus	1.9151	b cell	1.9835	3c protease	1.9878
Clinical characteristics	1.9138	Vaccine candidates	1.9835	tgev infection	1.9878
Mass spectrometry	1.9138	Host species	1.9833	cs dna	1.9878
National natural	1.9138	Respiratory viral infections	1.9832	Risk perception	1.9875
Rift valley	1.9138	Endothelial cells	1.9829	s1 protein	1.9875
Science china	1.9138	Sequence data	1.9829	Ring vaccination	1.9875
Valley fever	1.9138	DNA viruses	1.9826	Syrian hamster	1.9873
Respiratory virus infections	1.913	Host innate	1.9826	Wild mice	1.9873
Syndrome coronavirus	1.9096	Parainfluenza virus	1.9824	Yellow fever	1.9873
Classical swine fever virus	1.9087	Tract infections	1.9822	Climate change	1.9873
b cells	1.9074	South Korea	1.9821	Public health services	1.9873
Host response	1.9074	Acute respiratory infection	1.9817	Index patients	1.9872
Science foundation of china	1.9074	Reproduction number	1.9816	Small rna	1.9872
Viral proteins	1.9074	Surveillance system	1.9816	IC activity	1.9871
Virus disease	1.9065	Causative agent	1.9813	Ebola virus disease	1.9868
Clinical infectious diseases	1.9048	Multiple sclerosis	1.9811	RNA chaperone	1.9867
World health organization	1.9048	rsv infection	1.9809	Caco-2 cells	1.9867
Antiviral agents	1.9001	Cellular proteins	1.9808	m2 channel	1.9865
Cell culture	1.9001	West nile virus	1.9806	Overlapping genes	1.9865
Pulmonary disease	1.9001	Respiratory diseases	1.9805	Nasal mucosa	1.9865
Study protocol	1.9001	tgev infection	1.9805	Hepatitis e	1.9865
Dengue virus	1.8946	e protein	1.9802	Genetic drift	1.9865
Public health	1.893	Gene expression	1.9801	a7 gfp	1.9865
RNA replication	1.8915	Structural proteins	1.9799	Tumor cells	1.9864
Japanese encephalitis	1.8902	Acute respiratory tract	1.9792	Tanguticum nanoparticles	1.9864
Syndrome coronavirus infection	1.8864	Hand hygiene	1.9792	cfu ml	1.9864
Human respiratory syncytial virus	1.8841	Disease transmission	1.9788	Ward closure	1.9861
Synonymous codon usage	1.8824	Human rhinovirus	1.9785	Case definitions	1.9861
Clinical infectious	1.8813	Bacterial infections	1.9781	Richards model	1.9861
Health organization	1.8813	Cancer cells	1.9781	Epimedium koreanum	1.9861
Severe pneumonia	1.8813	DNA vaccines	1.9777	ms2 plp	1.986
Dengue virus infection	1.8772	Type III	1.9777	Gene therapy	1.9859
Clinical samples	1.8768	Viral pathogenesis	1.9773	Integrin b3	1.9859
Classical swine fever	1.8744	Zoonotic diseases	1.9773	Cardiovascular diseases	1.9859
Human antibody	1.869	Early detection	1.9765	Fourth site	1.9859
Lassa virus	1.869	Lung cancer	1.9756	Serial interval	1.9858
Pilot study	1.869	Nile virus	1.9756	trm cells	1.9858
Avian influenza viruses	1.8667	Human disease	1.9751	Electronic supplementary material	1.9857
Human respiratory syncytial	1.8667	rnase l	1.9751	Emergency nurses	1.9856
International health regulations	1.8667	Health systems	1.9746	Pet substrate	1.9856
Hepatitis c virus infection	1.8661	Incubation period	1.9746	fcov type	1.9856
Infectious bronchitis virus strain	1.8661	Rabies virus	1.9746	s1 text	1.9856
Vaccine development	1.8601	Adaptive immunity	1.9741	Global health research	1.9854
Protects hepatocytes from type I	1.8564	Multiplex pcr	1.9741	ace2 activity	1.9853
Type I interferon signaling disrupts	1.8564	nk cells	1.9741	$\beta$ 6 ko	1.9853
Adaptive immunity	1.8538	Feline coronavirus	1.9735	Global health	1.9852
Adenovirus type	1.8538	Human populations	1.9735	Ham tsp	1.9851
Nonhuman primates	1.8538	Common cold	1.9723	Blood culture	1.9849

**Table 8:** Best ranked terms extracted from Paper1 using C-Value

C-Value					
Title Corpus		Abstract Corpus		Content Corpus	
Terms	Rank	Terms	Rank	Terms	Rank
Respiratory syndrome	386.7309	Public health	1393.182	t cells	2063.1457
Virus infection	366.1263	Respiratory syndrome	1095.2091	Public health	1644.7156
Porcine epidemic diarrhea virus	329.7138	Infectious diseases	952.5625	Amino acid	1409.82415
Porcine epidemic diarrhea	318.0	Immune response	908.1835	Immune response	1400.94835
Epidemic diarrhea virus	306.0	Immune responses	841.6151	Influenza virus	1185.8689
East respiratory syndrome	284.0	Influenza virus	841.6151	Immune responses	1056.536
Middle east	261.5188	t cells	803.576	t cell	1056.37753
Epidemic diarrhea	256.7639	Virus infection	760.7811	Gene expression	1050.6716
Diarrhea virus	245.6692	Respiratory tract	727.4978	Viral replication	1021.5083
Infectious diseases	245.6692	Vviral infection	668.8542	Infected cells	939.72426
Respiratory syndrome coronavirus	240.0	Viral replication	665.6843	Cell lines	897.4057
Influenza a	225.0647	Viral infections	640.3249	Viral infection	888.6884
Public health	209.2151	East respiratory syndrome	638.0	Virus infection	872.68035
Syndrome coronavirus	191.7805	Respiratory syndrome coronavirus	636.0	Amino acids	866.816
Porcine epidemic	190.1955	Middle east	630.8151	mg ml	824.4975
Influenza virus	182.2707	Gene expression	627.6452	Infectious diseases	822.27855
Respiratory tract	180.6857	Infectious disease	613.3805	Present study	812.45177
Middle east respiratory syndrome	174.1446	RNA viruses	603.8707	Respiratory tract	812.13477
Middle east respiratory	170.0	Present study	575.3414	Epithelial cells	759.03855
Respiratory syncytial virus	166.0	Respiratory viruses	551.567	Previous studies	732.41119
Infectious bronchitis	160.0812	Acute respiratory syndrome	516.0	Room temperature	714.3426
Infectious disease	156.9113	t cell	513.5279	Cell culture	673.60907
Infectious bronchitis virus	156.0	Syndrome coronavirus	511.9429	Additional file	657.75946
East respiratory	136.3068	Porcine epidemic diarrhea	506.0	Viral infections	635.72848
Syncytial virus	134.7218	95percent ci	502.4331	Immune system	617.97689
Avian influenza	131.5519	Viral rna	499.2632	Respiratory syndrome	617.3429
Respiratory viruses	131.5519	Amino acid	489.7534	Cell line	611.16155
East respiratory syndrome coronavirus	130.028	Respiratory syncytial virus	472.0	Infectious disease	607.04063
Middle east respiratory syndrome coronavirus	129.2481	Cell lines	443.7895	$\mu$ g ml	576.13388
Influenza a virus	126.0	Respiratory infections	426.3549	Western blot	568.36754
Bronchitis virus	125.212	Epithelial cells	424.77	rnase l	565.0391
Respiratory infections	125.212	Virus replication	420.0151	Virus replication	560.6012
Systematic review	125.212	Polymerase chain reaction	408.0	Cell surface	543.9591
Ebola virus	120.4572	Epidemic diarrhea virus	406.0	xx	542.0572
Acute respiratory	117.2872	Epidemic diarrhea	402.5805	Host cell	539.83825
Viral infections	117.2872	Host cell	396.2406	Codon usage	523.03765
Virus replication	115.7023	Syncytial virus	378.806	Viral proteins	520.6601
Open access	109.3624	Porcine epidemic diarrhea virus	376.1524	Respiratory viruses	515.4298
Zika virus	109.3624	Antiviral activity	374.0512	nk cells	503.2256
Respiratory tract infections	102.0	Risk factors	374.0512	Time points	497.8367
Viral infection	101.4376	Immune system	369.2963	Influenza viruses	492.7648
Immune response	99.8526	Ebola virus	364.5414	Important role	491.0213
Hepatitis c virus	98.0	Chain reaction	355.0316	Allergic rhinitis	486.5835
Gene expression	96.6827	Influenza viruses	348.6918	Antiviral activity	481.3531
Pandemic influenza	96.6827	Infected cells	347.1068	Global health	473.9038
Respiratory syndrome virus	96.0	Diarrhea virus	340.7669	mg kg	470.0998
Epithelial cells	95.0978	Host cells	334.4271	Frequency domain	469.1489
Complete genome	93.5128	Important role	331.2572	Control group	466.13749
Syndrome virus	93.5128	Phylogenetic analysis	331.2572	Viral load	465.34499
Virology journal	93.5128	Polymerase chain	331.2572	Binding site	459.6391
Hepatitis c	91.9278	Respiratory disease	326.5023	Expression levels	453.6162
Immune responses	90.3429	Avian influenza	324.9173	Hong Kong	450.7237
Genome sequence	88.7579	Respiratory tract infections	320.0	Clinical signs	448.8613
Dengue virus	87.1729	Infectious bronchitis	285.2933	Protein expression	448.2274
Molecular sciences	84.0029	Cell culture	272.6136	Wild type	446.7833
Type i	84.0029	Hepatitis c virus	268.0	Endothelial cells	441.4120
Acute respiratory syndrome	84.0	Health care	264.6887	Table s1	438.4006
Complete genome sequence	84.0	Zika virus	264.6887	Flow cytometry	437.4496
Human coronavirus	82.4181	Infectious bronchitis virus	260.0	Saudi Arabia	433.4872
Respiratory infection	82.4181	Tract infections	258.3489	Viral genome	433.3992
Case report	80.8331	Hepatitis c	255.179	Negative control	433.2230
Tract infections	80.8331	Innate immune response	252.0	–	431.7890
Risk factors	79.2481	Monoclonal antibodies	248.8391	Cell types	431.1098
Spike protein	77.6632	Viral genome	247.2542	Viral entry	427.9399
t cell	77.6632	Type I	242.4993	Cell death	425.24544
Acute respiratory infections	76.0	Central nervous system	242.0	er stress	423.185
Coronavirus infection	74.4932	Amino acids	239.3293	Significant differences	420.6490
RNA viruses	74.4932	Animal models	237.7444	Health care	420.4905
Severe acute respiratory	72.0	Real-time pcr	236.1594	Tcid 50	417.3734
Sars coronavirus	71.3233	Dengue virus	232.9895	Cathepsin l	410.5053
Isothermal amplification	69.7384	Viral load	232.9895	Risk factors	408.9203
Respiratory disease	69.7384	World Health Organization	232.0	Positive selection	405.7504
BMC public health	66.0	Cell line	231.4045	Cell cycle	400.9955
Disease virus	64.9835	Viral proteins	229.8196	Nucleotide sequences	397.8256
t cells	63.3985	Nervous system	226.6496	Plasma membrane	393.5990
Influenza viruses	61.8135	Wide range	223.4797	Intensive care	392.2782
Acute respiratory infection	60.0	Virus infections	221.8948	Host cells	384.82889
Type i interferon	60.0	Middle east respiratory syndrome	220.5832	Hand hygiene	383.5609
Journal frontiers	58.6436	Immunodeficiency virus	218.7248	Significant difference	382.6099
Fever virus	57.0587	Spike protein	218.7248	Immune cells	381.02498
Respiratory syncytial	57.0587	Life cycle	217.1399	Reference genes	380.3909
Severe acute	57.0587	Recent years	217.1399	HIV aids	377.2211
Respiratory tract infection	56.0	Codon usage	215.5549	Avian influenza	376.8688
Antiviral activity	55.4737	Viral pathogens	215.5549	Serum samples	375.8625
BMC infectious	55.4737	Pandemic influenza	213.9699	Body weight	375.0021
Hong Kong	55.4737	Clinical signs	212.385	Fig. 1a	374.0511
Viral replication	55.4737	Dendritic cells	209.2151	Membrane fusion	374.0511
Virus infections	55.4737	Acute respiratory syndrome coronavirus	208.9735	Clinical trials	373.8750
BMC infectious diseases	54.0	Bronchitis virus	207.6301	Time point	373.3719
Respiratory viral infections	54.0	Endoplasmic reticulum	207.6301	Protein synthesis	369.2962
Case study	53.8887	RNA virus	207.6301	Dengue virus	367.7113
Dendritic cells	53.8887	Saudi Arabia	207.6301	e protein	367.7113
Mini review	53.8887	Innate immunity	206.0451	High levels	365.3339
RNA virus	53.8887	Recent studies	206.0451	Virus particles	364.5414
Transmissible gastroenteritis	53.8887	Economic losses	204.4602	Target cells	362.5601
BMC public	52.3038	Porcine epidemic	204.4602	Viral particles	360.4204
Monoclonal antibodies	52.3038	World health	204.4602	Dendritic cells	357.5675
Creative commons cc-by 4	51.0824	Global health	202.8752	Total number	356.4580
Influenza pandemic	50.7188	Type 1	202.8752	Cancer cells	356.0883
Type 1	50.7188	Vaccine development	201.2902	Disease control	355.2957

**Table 9:** Expanded terms from Table 6

sous_corpus	Measure	Term	Domain relevant	COVID-19 surveillance	Syndromic surveillance	Incomplet disease name
title	C-value	Respiratory syndrome coronavirus	n	n	n	y
		Porcine epidemic diarrhea	y	n	n	n
		Syndrome coronavirus	n	n	n	y
		Epidemic diarrhea virus	n	n	n	y
		Acute respiratory syndrome	n	n	n	y
		Public access	n	n	n	n
		Diarrhea virus	n	n	n	y
		Infectious bronchitis	y	n	y	n
		Acute respiratory	n	n	n	y
		Bronchitis virus	y	n	y	n
	F-TFIDF-C	Journal pre-proof	n	n	n	n
		Virology journal	n	n	n	n
		Influenza pandemic	y	n	n	n
		Coronavirus spike	y	y	n	n
		BMC public health	n	n	n	n
		Influenza virus infection	y	n	n	n
		Emerging infectious	y	n	y	n
		Prcine circovirus type	n	n	n	y
		Codon usage	n	n	n	n
		Respiratory syndrome	n	n	n	y
abstract	C-value	Acute respiratory syndrome	n	n	n	y
		Respiratory syndrome coronavirus	n	n	n	y
		East respiratory syndrome	n	n	n	y
		Syndrome coronavirus	n	n	n	y
		Present study	n	n	n	n
		Chain reaction	n	n	n	n
		Syncytial virus	n	n	n	y
		Porcine epidemic diarrhea	y	n	n	n
		Polymerase chain	n	n	n	n
	F-TFIDF-C	Virus infections	y	n	y	n
		Porcine epidemic	n	n	n	y
		Clinical samples	n	n	n	n
		Codon usage	n	n	n	n
		Mers-cov infection	y	y	n	n
		Pandemic influenza	y	n	n	n
		Viral entry	y	n	y	n
		95 percent confidence interval	n	n	n	n
		Immune cells	n	n	n	n
		Influenza pandemic	y	n	n	n
		Sono stati	n	n	n	n
content	C-value	Infected cells	n	n	n	n
		Respiratory syndrome	n	n	n	y
		Present study	n	n	n	n
		Individual components	n	n	n	n
		Essential medicines	n	n	n	n
		Previous studies	n	n	n	n
		de los	n	n	n	n
		Functional task	n	n	n	n
		der Schwangerschaft	n	n	n	n
	F-TFIDF-C	Health emergency	y	n	y	n
		Membrane rafts	n	n	n	n
		pcr products	n	n	n	n
		afa dr	n	n	n	n
		COD trypsin	n	n	n	n
		2c atpase	n	n	n	n
		Naked mole	n	n	n	n
		Intracellular delivery	n	n	n	n
		Close contact	y	n	y	n
		Final dataset	n	n	n	n
		Respiratory syndrome	n	n	n	y
title + abstract	C-value	Acute respiratory syndrome	n	n	n	y
		Respiratory syndrome coronavirus	n	n	n	y
		East respiratory syndrome	n	n	n	y
		Syndrome coronavirus	n	n	n	y
		Syncytial virus	n	n	n	y
		Porcine epidemic diarrhea	y	n	n	n
		Antiviral activity	y	n	n	n
		Acute respiratory syndrome coronavirus	n	n	n	y
		Infectious bronchitis	y	n	n	n

Each term has been evaluated by an expert according 4 criteria: domain relevant, COVID-19 surveillance, syndromic surveillance, incomplet disease name (y: yes, n: no)

**Table 10:** 60 terms randomly selected from FASTR variants (Section The driven term extraction approach)

Influenza virus	Evaluation	Respiratory infections	Evaluation	Infectious diseases	Evaluation
Influenza a/wsn/33 virus	Not relevant	Respiratory virus infections	Relevant	Diseases relates to infectious	Relevant
Viruses and conventional influenza	Relevant	Respiratory viral infection	Relevant	Disease called feline infectious	Relevant
Virus remains the influenza	Lack of context	Infection by respiratory	Relevant	Infectious animal diseases	Relevant
Influenza by virus	Not relevant	Infections of the respiratory	Relevant	Infectious enteric diseases	Relevant
Influenza vaccine virus	Relevant	Infect respiratory	Relevant	Disease without being infectious	Not relevant
Virus and canine influenza	Relevant	Infections are respiratory	Relevant	Disease has an infectious	Lack of context
Virus influenza	Relevant	Infected with respiratory	Relevant	Infectious disease	Relevant
Viruses such as influenza	Relevant	Infection with other respiratory	Relevant	Disease named it infectious	Lack of context
Influenza b viruses	Relevant	Respiratory virus infection	Relevant	Infectious swine diseases	Relevant
Viruses and emerging influenza	Relevant	Infection transmitted via respiratory	Relevant	Disease models for infectious	Lack of context
Viral infections	Evaluation	Sars coronavirus	Evaluation	Incubation period	Evaluation
viral bronchopulmonary infection	Relevant	Coronavirus is urbani sars	Not relevant	Incubating period	Relevant
Virally infected	Relevant	Coronavirus of 18 sars	Not relevant	Periods of incubation	Relevant
Viral respiratory infections	Relevant	Coronavirus that causes sars	Relevant	Incubation periods	Relevant
Infection and encounter virally	Lack of context	Coronavirus named sars	Relevant	Period of incubation	Relevant
Infection or viral	Lack of context	Coronavirus related to sars	Relevant	Period than incubation	Lack of context
Viral skin infection	Relevant	Coronavirus isolated from sars	Relevant	Period and incubation	Lack of context
Virals infection	Relevant	Coronavirus responsable du sars	Relevant	Incubation for period	Lack of context
Infection with one viral	Relevant	Sars -associated coronavirus	Relevant	Period and incubating	Lack of context
viral opportunistic infections	Relevant	Sars human coronavirus	Relevant	Period covering an incubation	Relevant
infection at high viral	Lack of context	Sars and coronavirus	Relevant	Period of extrinsic incubation	Relevant

## Appendix B

See Figs. 10 and 11.

## Footnotes

1 https://www.semanticscholar.org/cord19/download

2 https://code.google.com/p/maui-indexer/.

3 https://pypi.python.org/pypi/topia.termextract/1.1.0.

4 http://www.nzdl.org/Kea/.

5 https://padi-web.cirad.fr/en/.
